# New through-the-needle brush for pancreatic cyst assessment: a randomized controlled trial

**DOI:** 10.1016/j.igie.2023.08.006

**Published:** 2023-08-29

**Authors:** Filipe Marques, Igor Schliemann, Wouter van der Wijngaart, Urban Arnelo, Niclas Roxhed, Francisco Baldaque-Silva

**Affiliations:** 1 KTH Royal Institute of Technology, Micro and Nanosystems, Stockholm, Sweden; 2 Pathology and Cytology Department, Karolinska University Hospital Stockholm, Sweden; 3 Department of Surgical and Perioperative Sciences/Surgery, Umeå University, Umeå, Sweden; 4 Division of Surgery, CLINTEC, Karolinska Institute, Stockholm, Sweden; 5 MedTechLabs, Bioclinicum, Karolinska University Hospital, Solna, Sweden; 6 Advanced Endoscopy Center Carlos Moreira da Silva, Gastroenterology Department, Pedro Hispano Hospital, Matosinhos, Portugal; 7 Department of Medicine, Karolinska Institute, Stockholm, Sweden; 8 Center for Upper Gastrointestinal Diseases, Karolinska University Hospital, Stockholm, Sweden

## Abstract

**Background and Aims:**

Current EUS technologies are suboptimal in the assessment of pancreatic cystic lesions (PCLs). We developed a new through-the-needle brush, the “loop brush,” to improve the cellular yield, and thereby sensitivity, of EUS-guided FNA (EUS-FNA) of pancreatic cysts. In this study, we evaluated its safety and efficacy.

**Methods:**

We performed an in vivo randomized controlled trial in pigs using artificial cysts. In 1 group, the loop brush was deployed through a 22-gauge needle using EUS-FNA into the cysts. In the control group, cystic puncture was performed with a standard needle. Loop brushes were visually inspected after the procedure. Cytologic assessment, cell counting, and hemoglobin analysis were performed in samples from both groups.

**Results:**

One hundred fourteen artificial cysts were punctured in 6 pigs, 57 in each group. Neither adverse events nor significant device malfunction occurred during loop brushing. Samples collected with the brush had nondetectable concentrations of hemoglobin in 72% of cases (41/57), and 26% (16/57) had <.6 g/dL, with no significant differences with the controls (*P* = .32). Brushing cell counts were associated with significantly increased cell counts (11.7 times the median difference, *P* < .0001). Cytologic smears were diagnostic in 77% of cases in the brushing group and 54% in the control group (*P* = .01 [Fisher exact test]; *P* = .006 [χ^2^ test]).

**Conclusions:**

The procedure using the new loop brush appears to be safe, causing neither significant bleeding nor device malfunction. Samples obtained with the loop brush were suitable for cytologic analysis and showed significantly higher cell yield than controls. Further clinical studies are warranted.

Pancreatic cancer remains a critical disease globally because of its extremely aggressive nature and poor survival rate.^1^^,^^2^ The low success rate of treatment results from an advanced disease at the time of diagnosis.^3^ Pancreatic cystic lesions (PCLs) are precursors of a significant proportion of pancreatic cancers and have a prevalence of up to 49% in the adult population.^4^^,^^5^ Considering that most cysts are benign, it is important to detect those with malignant potential to ensure proper follow-up or treatment.^6^ Radiologic cross-sectional imaging lacks accuracy for differential diagnosis because no clear pathognomonic features exist for specific cyst types.^7^^,^^8^

EUS-guided fine-needle aspiration (EUS-FNA) of cystic lesions followed by cytologic analysis has been used to differentiate benign, potentially malignant, and malignant pancreatic cysts, albeit with dismal results.^9^ The collected cystic fluid from EUS-FNA often presents little to no cell yield and frequently shows GI contamination, resulting in relatively low sensitivity.^10^ Acellularity has been reported in up to 65% of cases, which explains the low diagnostic accuracy of associated cytologic analyses, which is as low as 31%.^8^^,^^11^ Studies show that the diagnostic yield of a cyst can be significantly increased when cells are retrieved directly from the cystic wall.^12^^,^^13^ Current sampling technologies such as Moray microforceps (Steris Corporation, Mentor, Ohio, USA) and EchoBrush (Cook Endoscopy, Bloomington, Ind, USA) aim to increase the diagnostic yield of EUS. However, these devices might be too damaging for PCLs and may be associated with significant adverse events.^14^^,^^15^

Previously, we developed and tested,^16^ in vitro and ex vivo, a new loop-shaped minimally invasive nitinol-based cell brush, the “loop brush,” for cytologic diagnosis of pancreatic cysts. This device is designed to detach cells from the cyst wall into the cystic fluid, followed by aspiration of the fluid. Unlike traditional cytologic brushes, cells are not expected to attach to the brush and are instead collected by aspirating the liquid content of the cysts. The loop brush can be operated through a 22-gauge FNA needle in conjunction with EUS. After insertion into the cyst, the loop brushes the inner cystic wall, followed by aspiration of the cyst’s liquid content. In ex vivo tests, the cell content in fluid retrieved from cyst models and a bovine ovary cyst was significantly increased after brushing. The fluid viscosity was also simulated with water and glycerol (1000 times more viscous than water) and showed no hindrance to the loop brush procedure. No adverse events, such as cyst perforations or device malfunctions, were observed. In this in vivo randomized controlled trial (RCT) in pigs, we tested the cell yield and safety profile of this new loop brush using artificial cysts, comparing this new device with the standard procedure, EUS-FNA.

## Methods

### Study design

We performed an RCT using 6 Yorkshire-Swedish farm pigs (2 males and 4 females, weighing 34.5-43 kg). Allocation sequencing was performed by randomizing experiments with an equal number of controls and loop brushes using a random sequence generator. After cyst identification by EUS, only 1 engineer had access to the allocation sequence and provided the devices (either FNA with a loop brush or conventional FNA) to the endoscopist accordingly.

A single needle pass was performed for all controls and loop brush procedures. The aspirated fluid content from each cyst was analyzed cytologically with rapid on-site evaluation (ie, a droplet of aspirate was passed directly from the FNA needle to a glass slide and prepared on-site). The cytologist was blinded with respect to the device used (loop brush vs conventional FNA). Loop brushes were visually inspected and scrutinized for damage after each procedure. The small and smooth surface area of the loop brush hampers cell attachment to the brush, so no component of the loop brush was used for cytology analysis. Two cysts were analyzed histologically (1 brushing, 1 control). Hemoglobin, a surrogate marker for bleeding, and cell concentration of the cystic fluid were also analyzed. Hemoglobin analysis was performed with a HemoCue HB system (HemoCue AB, Ängelholm, Sweden), and cell concentration analysis was performed with a Countess II FL automated cell counter (Thermo Fisher Scientific Inc, Uppsala, Sweden).

Details on surgical cyst preparation, cytologic and histologic analysis, and hemoglobin and cell counting analysis can be found in Appendix parts 1 to 3 (available online at www.igiejournal.org), respectively. Cysts with clear food debris, here described as sustenance debris, were excluded from the cell counting analysis. We performed 20 additional procedures with the loop brush in 2 additional pigs (2 males, weighing 36 kg each) to further assess the procedural safety of this device (Appendix part 4, available online at www.igiejournal.org).

### Loop brush

**Figure 1:**
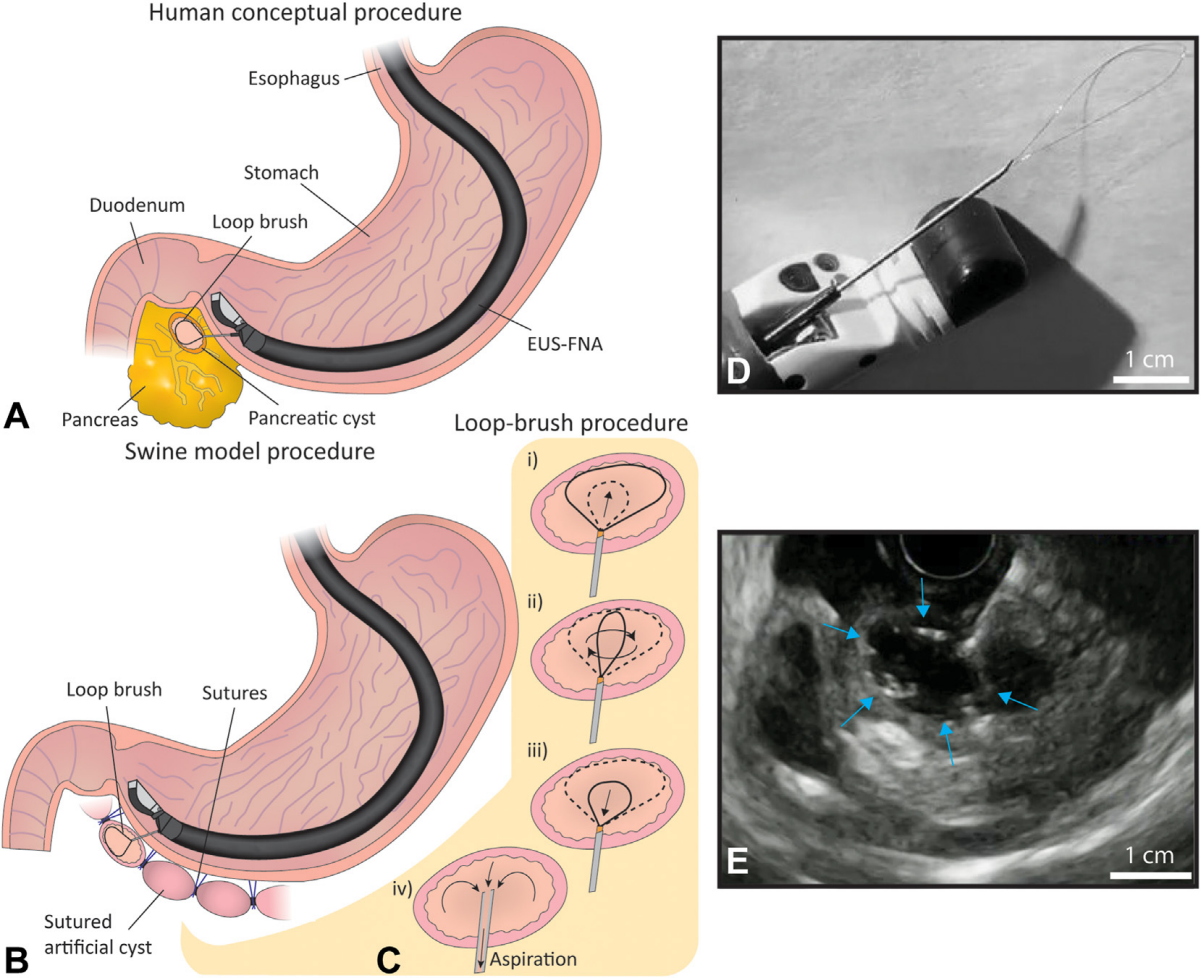
**A,** Conceptual illustration of the EUS-FNA procedure on a human pancreatic cyst, coupled with the loop brush. **B,** Pig model procedure of EUS-FNA procedure on artificial cysts, coupled with the loop brush. The artificial cysts are surgically prepared using the small intestine and sutured to the antrum or body of the stomach. **C,** The loop brush procedure showing cyst puncture: *i)* introduction and expansion of the brush into the cyst; *ii)* rotation of the brush against the cystic wall; *iii)* removal of the brush from the cyst; and *iv)* aspiration of the liquid content of the cyst. **D,** A loop brush inserted in an FNA needle through a linear echoendoscope. **E,** US image of a loop brush (*arrows*) during EUS-FNA in a cyst.

The loop brush is a single-use device for operation through a 22-gauge FNA needle. The device is designed to brush cells from human pancreatic cysts during EUS-FNA (Fig. 1A). The brush has 2 main components. The first component is a 50-μm diameter nitinol wire (NiTi #1 wire, .05 mm straight annealed light oxide; Fort Wayne Metals, Castlebar, Ireland) bent into a 2-cm diameter loop and glued to the distal end of a 250-μm diameter nitinol guidewire (NiTi #1 wire, .25 mm straight annealed light oxide; Fort Wayne Metals) with medical-grade Loctite 4011 (Henkel Corp, Düsseldorf, Germany). The second component is a protective sheath made of Teflon (221657; Zeus Inc, Orangeburg, SC, USA) or polyimide (.38 μm inner diameter and .4 μm outer diameter tubing; Zeus Inc) that prevents damage to the loop brush during brush retrieval.

We first attempted to use a protective sheath made of Teflon. However, the Teflon sheath tended to break easily if not retrieved slowly from the FNA needle, making it cumbersome to operate. Therefore, we changed the protective sheath material to polyimide.

### Cyst model

Because pancreatic cysts are not commonly found in large laboratory animals, we mimicked this condition in pigs by creating 2-cm-sized vesicles from the jejunum, called “cysts” in this article. These cysts were sutured to the backside wall of the stomach, mimicking the position of a cyst in the pancreas (Fig. 1B). Structurally, the cysts possessed a single cavity filled with lukewarm water and no solid components.

### EUS-FNA with and without the loop brush

EUS-FNA was performed with a linear echoendoscope (GF-UCT140-AL5; Olympus, Tokyo, Japan) coupled to an Aloka ProSound Alpha 10 (Hitachi Aloka Medical, Tokyo, Japan). Before the EUS procedure, after a gastroscopy, a Guardus gastric overtube (Kungshusen Medicinska, Mariefred, Sweden) was placed and positioned into the distal esophagus to enable easier insertion of the EUS instrument. Control procedures were performed with the standard EUS-FNA technique using an Expect Slimline EUS aspiration 22-gauge needle (Boston Scientific, Helsingborg, Sweden). Brushings were performed using the same brand of FNA needles with a preloaded loop brush inside the needle. A new FNA needle with or without a new loop brush was used for each function.

Standard EUS-FNA was performed by puncturing the cyst, aspirating 3 to 6 mL of cyst fluid, followed by needle removal from the cyst. The brushings proceeded with the following subsequent steps: the cyst was punctured; the protective sheath (Teflon in 39 procedures in pigs 1, 2, 3, and 6; polyimide in 18 procedures in pigs 4 and 5) and brush were advanced 5 mm through the needle into the cyst; the brush was inserted 3 cm into the cyst through the needle and the protective sheath, wherein the deployed brush obtained its loop-like shape that conformed to the cystic wall (Fig. 1C, i)); the loop brush handle was repeatedly rotated 3 times clockwise and 3 times counterclockwise at approximately 1 rotation per second for a total of 1 minute (Fig. 1C, ii)); the protective sheath was maintained inside the cyst while the loop was retracted from the cyst (Fig. 1C, iii)); the loop brush and sheath were completely removed from the needle; a syringe was attached to the Luer lock of the FNA needle; and 3 to 6 mL of cyst fluid were aspirated following the standard EUS-FNA procedure.

The loop brush was fully integrated with the EUS-FNA needle, as shown in Figure 1D. Videos 1 and 2 (available online at www.igiejournal.org) show the cyst during EUS-FNA with a loop brush in operation, including the wire loop (hyperechoic) turning, appearing and disappearing from the focal plane of the echoendoscope (Fig. 1E), and retrieval of the brush and protective sheath from the FNA needle after 1 minute.

### Sample size calculation

A Mann-Whitney rank test sample size calculation was performed at 95% power and a Type I error rate (α) of 5% to detect a median difference of 500,000 cells/mL between samples acquired using standard EUS-FNA and EUS-FNA with a loop brush. Pooled standard deviation was 800,000 cells/mL (estimated based on prior cell counting results),^16^ indicating a sample size of 38 cysts per group.

A previous study^17^ showed an adverse event rate of EUS-guided fine-needle biopsy sampling to be 1.2% in 70 procedures. To provide the first safety assessment of the loop brush, we compared the adverse event rate of EUS-guided fine-needle biopsy sampling with our device. Accordingly, we planned at least 8 cysts per group per animal, but 29 more cysts (including 20 devices used in Appendix 4) were created to test safety further and account for possible device malfunctions.

### Ethics

This study was approved by and carried out in accordance with the Swedish Agricultural Agency (ethical application registration number 605-2021) and Karolinska Institutet regulations. All procedures were conducted per Directive 2010/63/EU and Council Directive 98/58/EC and applicable institutional, national, and international guidelines and ethical standards. The study was designed and implemented and the manuscript written in accordance with the Animal Research: Reporting of In Vivo Experiments^18^ and Consolidated Standards of Reporting Trials^19^ guidelines (see annexed checklists) on reporting of in vivo experiments in animals and RCTs, respectively.

### Endpoints

All data followed intention-to-treat analysis. Loop brush use was tested and categorized into successful and unsuccessful using specific criteria. A successful investigation included all the following: deploying the brush inside the cysts, rotating the brush against the cystic wall, removing the brush from the FNA needle, and removing the liquid content of the cyst. If ≥1 successful criteria failed, then loop brush use would be deemed unsuccessful.

Loop brush safety was assessed. Adverse events were considered according to the American Society for Gastrointestinal Endoscopy severity criteria.^20^ Given the nature of this study, these adverse events were the device breaking inside a cyst with device parts remaining in the cyst and perforation and bleeding, specifically the presence of macroscopic blood in collected samples. All cysts were collected after the procedure, opened, and carefully visually inspected for broken brush components, bleeding, and/or perforation.

Hemoglobin analysis of aspirated samples assessed bleeding in the cyst. Cell count analysis assessed cell acquisition capability with and without the loop brush procedure. Histology determined the tissue structural variations after the loop brush procedure. Cytologic smears were assessed by their appropriateness to provide a diagnosis, specifically adequate samples with sufficient cellularity and cell integrity in the samples for a diagnostic assessment.

### Statistical analysis

Differences between both groups, regarding cell counting and hemoglobin analysis, were applied to a 1-sided Mann-Whitney rank test with a significance level of 5% to test for a statistically significant difference. Negative and positive outcomes using rapid on-site evaluation for both standard EUS-FNA and EUS-FNA with the loop brush were applied to a 2-sided Fisher exact test and χ^2^ test to calculate the odds ratio and *P* value for a significance level of 5%. The software used was GraphPad Prism 8 software (GraphPad Software, San Diego, Calif, USA).

## Results

**Figure 2:**
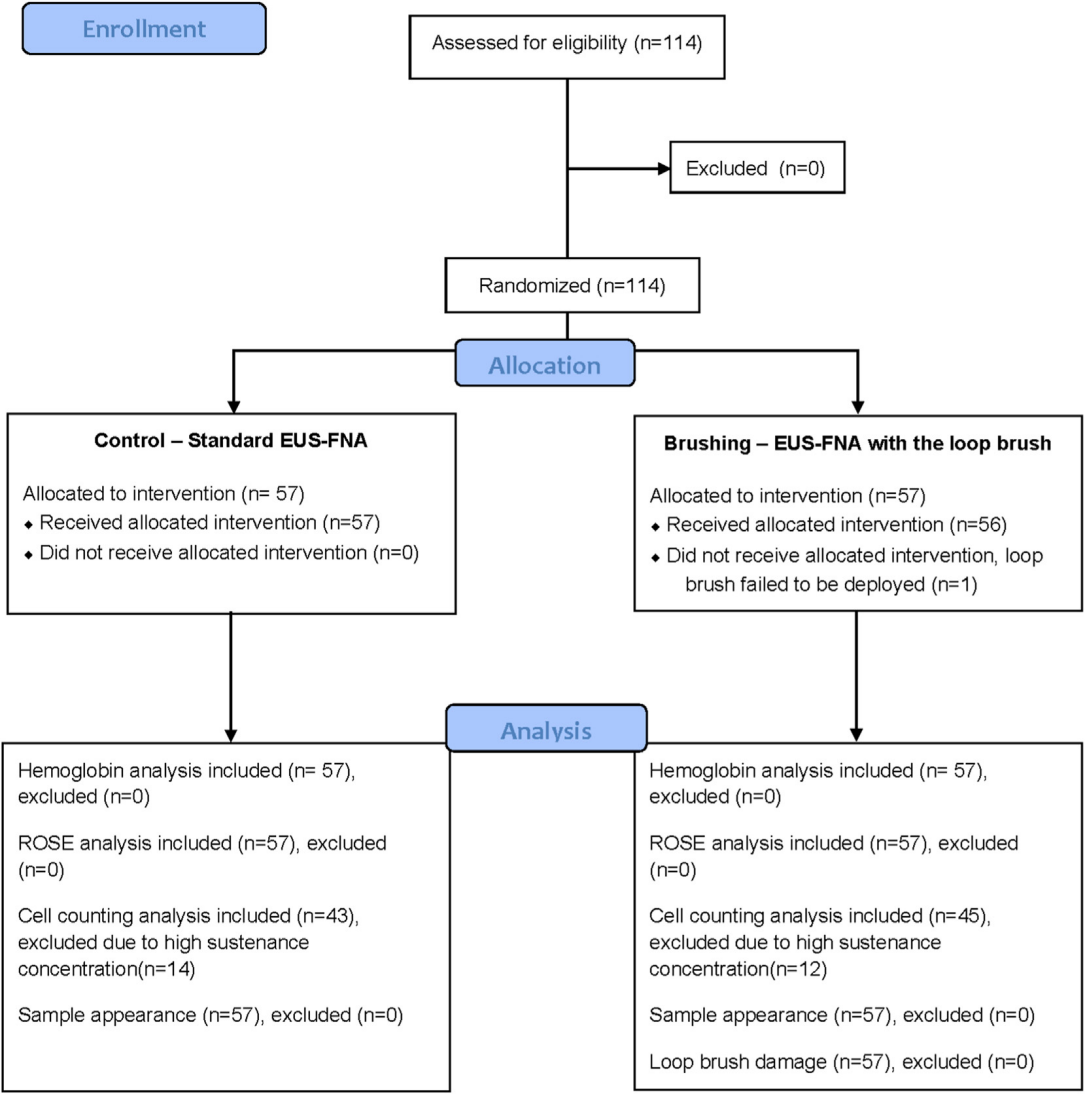
Study flowchart of standard EUS-FNA versus EUS-FNA with a loop brush in artificial cysts. *ROSE*, Rapid on-site evaluation.

We prepared 114 artificial cysts surgically and tested the procedural safety and diagnostic assessment of standard EUS-FNA (n = 57) compared with EUS-FNA with a loop brush (n = 57) (Fig. 2). Of the 57 loop brushes, 56 were used successfully, independently of the position of the cyst or the bending of the endoscope. One brushing was unsuccessful because the loop brush failed to be fully deployed into a cyst. However, this unsuccessful brush was easily pulled out from the cyst and was found intact in the postprocedure analysis.

**Figure 3:**
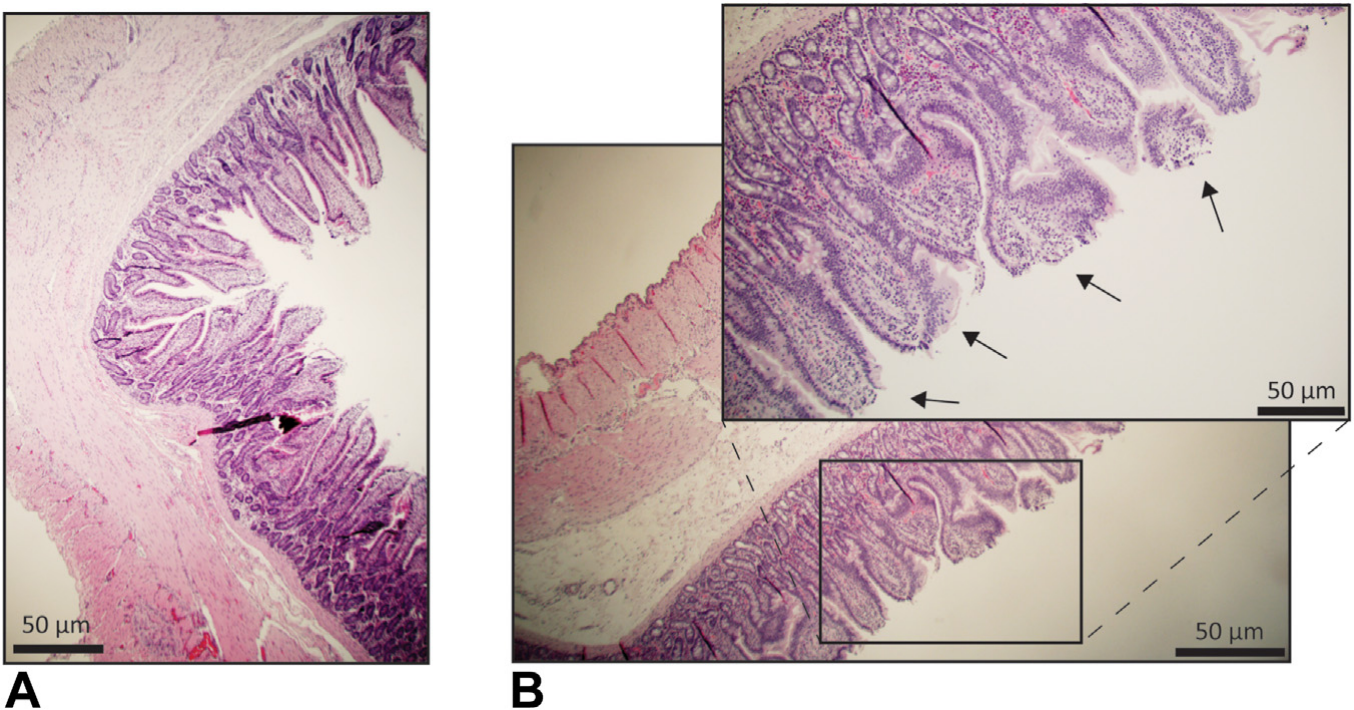
**A,** Four times magnification image of a control artificial cyst after histologic preparation. **B,** Four times and 10 times magnification images of an artificial cyst after loop brushing after histologic preparation. *Arrows* indicate regions of contact between the loop and the tissue.

On 2 occasions the Teflon sheath broke during removal from the FNA needle, yet all parts came out in full. Postmortem cyst inspection showed no adverse events. The polyimide sheath was easy to operate and showed no technical problems during operation. Hemoglobin concentrations indicated no significant difference (*P* = .32) between samples acquired after loop brushing and control procedures (Fig. 3A). Hemoglobin was not detectable on 72% of samples after loop brushing and on 79% of control samples. The highest hemoglobin concentration detected was .6 g/dL for loop brushings and .8 g/dL for controls.

Experiments generated 49 control and 50 brushing samples with a semitransparent and a slightly yellow appearance. Eight control and 7 brushing samples presented a semitransparent and orange appearance. No grossly bloody specimens were observed in either group.

Cell counting showed that samples obtained after brushing contained 11.7 times more cells compared with control samples. Statistical analysis indicated a significant difference (*P* < .0001) between brushing and control samples (Fig. 3B).

**Figure 4:**
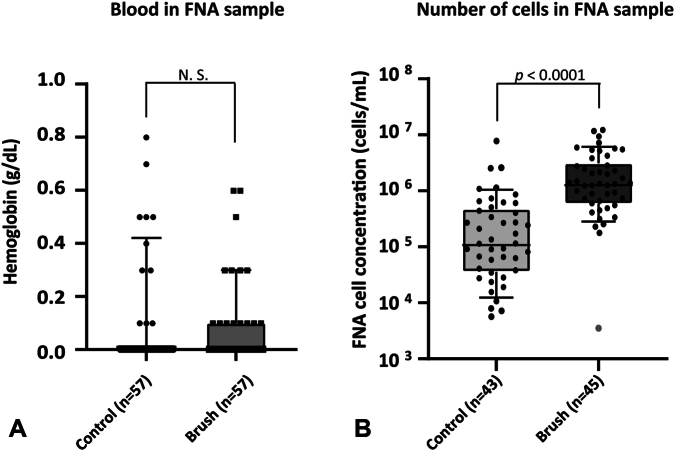
**A,** Box and whisker plot indicating the measured hemoglobin concentrations of 57 control samples and 57 loop brush FNA samples, showing no significant difference between controls and brushings (*P* = .32). **B,** Box and whisker plot showing the cell count results of control samples and loop brush samples from all pigs, indicating a significant difference (*P < .*0001) between brushing and no brushing. The *green dot* shows the value for the cyst in which the loop brush failed to be deployed. Whiskers indicate the 10th to 90th percentile.

The unsuccessful brush puncture can be seen as an outlier in the brushing results (Fig. 3B, dot in green), where 1 sample had a cell concentration of the same order of magnitude as that of the control samples. Fourteen control samples and 12 brushing samples were excluded from cell counting analysis because of excessive sustenance remains (not plotted). Histology analysis indicated that loop brushing removed cells from the top part of the villi (Fig. 4).

Samples were considered diagnostic in 77% of brushing cases versus 53% in control cases with a significant difference between the 2 groups (*P* = .01 [Fisher exact test]; *P* = .006 [χ^2^ test]). We identified cells from both the small intestine and dietary plants (resulting from sustenance debris), showing a sustenance layer still present in the walls of cysts (Appendix part 5, available online at www.igiejournal.org).

Appendix part 4 shows that all 20 additional loop brush procedures were successful. Postmortem cyst inspection showed no adverse events. Hemoglobin results showed that 75% of samples had nondetectable hemoglobin, and the highest concentration was .7 g/dL, concurrent with the data in the RCT. Cell concentrations of samples were in the same order of magnitude as samples in the RCT. No control procedures or rapid on-site evaluation was performed in this subset of cysts.

Appendix part 6 (available online at www.igiejournal.org) shows an overview of results for each individual pig. Cell counting showed that samples after brushing contained between 4.5 and 10.5 times more cells compared with control samples, relative to median values.

## Discussion

Currently, the diagnostic accuracy of EUS-FNA is as low as 31%.^8^^,^^11^^,^^21^ Through-the-needle biopsy sampling and brushing devices such as the Moray microforceps and the EchoBrush are integrated in 19-gauge FNA needles. The Moray microforceps has been used for histologic diagnosis of pancreatic cysts. However, this device samples a small portion of the cyst wall, and its use has been associated with severe adverse events.^15^ The EchoBrush was a cytologic brush used to increase the diagnostic yield of cystic samples.^22^ However, an increase in adverse events led to the discontinuation of this device.^14^ No through-the-needle biopsy sampling devices for 22-gauge FNA needles have been reported.

In the present study, a new through-the-needle loop brush was evaluated, developed to increase the cytologic yield of EUS in the diagnosis of PCLs. The loop brush aims to be minimally invasive and robust while still successful at brushing cells. Therefore, we chose a 22-gauge needle, with an acceptable trade-off between minimal invasiveness, flexibility, and procedural safety.

We successfully performed a preclinical assessment of the loop brush used during EUS-FNA in 8 pigs by surgically modifying the small intestine to mimic cysts in humans. No devices showed adverse events, neither on the cysts nor the animals. Sample hemoglobin concentrations, a surrogate marker of wall damage, revealed low levels of hemoglobin, with no significant difference with control samples. Cell counting indicated at least 4.5 times higher cell yields (median difference) in brush samples compared with control samples. Histology showed that the loop brush removed cells from the cyst inner wall with cystic damage. The cytologic smears showed that brush samples could be used for cytologic assessment with diagnostic samples in 77% of the brush group compared with 53% in the control group.

This study also showed that the Teflon sheath protects the brush from undesired damage but is too fragile for practical use. In the second version of our device, the change from Teflon to polyimide sheaths facilitated the retrieval of the loop brush from the needle lumen. In data presented in Appendix 6, the average cell counting ratio between the loop brush versus control, per pig, indicated similar differences between using Teflon or polyimide sheaths (4.5, 20.8, and 3.5 times with Teflon and 15.3 and 3.6 times with polyimide). Hemoglobin concentrations also remained at similar values independently of the sheath used.

The only unsuccessful brush deployment event was attributed to a too-thick glue seam that created considerable friction between the guidewire and the protective sheath component. The glue seam does not add more than .015 mm in radius to the brush, indicating a critical fabrication step to be taken into consideration on assembly of devices.

Our results showed that the loop brush can remove cells from living tissue without adverse events. The cytologic assessment indicated that loop brushing samples were of sufficient quality for cytology, preserving cell morphology for downstream diagnosis. The current study also indicated that adding the loop brush to EUS-FNA does not add short-term risks to the standard procedure. Thus, the risk assessment of the device might be translatable to humans.

This study does not test the loop brush in pancreatic cysts because of a lack of appropriate animal models. Pancreatic cysts can be reproduced in genetically modified mice, but whereas mice models could provide information on cyst formation, their size does not allow testing devices mimicking human conditions.^23^ Pigs represent the human anatomy scale and can be genetically modified to have solid lesions, but there are no known cystic models.^24^ Human pancreatic cysts can have a different cellular lining, with some intraductal papillary mucinous neoplasm showing intestinal-like cellular lining.^25^ Therefore, we created artificial cysts from the small intestine to produce and position cysts at will. These cysts are suboptimal for diagnostic yield evaluation, given the natural shedding of cells from the mucosa of the small intestine and the sustenance remnants with respect to puncturing pancreatic cysts. Our pig model does not replicate the liquid environment of PCLs, given all the biochemical components absent in our “cysts.” However, the loop brush can overcome even cyst fluid with relatively high viscosity, as shown during ex vivo tests.^16^ Our artificial cysts were all similar in size, and all punctures were performed through the stomach wall. This study was also conducted with 2 types of sheath material (Teflon and polyimide). Yet, as previously mentioned, cell counts and hemoglobin concentrations remained in similar ranges with either sheath material. Sustenance was found in several cysts, limiting their respective cytologic and cell counting analysis. The blood flow of neoplastic PCLs is also not modeled here. However, our artificial cysts were made of highly vascularized jejunum. Finally, our pig model cysts do not possess the same characteristics as real pancreatic cysts, so adverse events such as pain, pancreatitis, or infections are not shown. However, current animal models for EUS-FNA fail to reflect such events.

Diagnostic yield and cell counting results of brushed samples indicate the potential of the loop brush to abrade cells from live tissue and its potential use in the cytologic analysis. The pig model enabled the assessment of the loop brush procedure, where the endoscope and FNA needle are used in a very similar manner to what is done during common clinical practice. Given the similarities of our model to EUS-FNA in humans, the loop brush has the potential to increase the cellular yield of EUS-FNA for the diagnosis of pancreatic cysts. This would lead to a proper diagnosis of cysts as benign, potentially malignant, and malignant, improving the management of patients with PCLs. In this respect, the loop brush presents a promising alternative to current technologies, suggesting safety results similar to standard EUS-FNA and a comparatively higher diagnostic yield. Ultimately, the loop brush could increase treatment options and produce better, cost-effective care for patients. Clinical trials are needed to assess the diagnostic yield of our newly developed brush compared with conventional technologies. Further trials are also required to test the loop brush safety profile and the potential to increase the diagnostic yield in different kinds of pancreatic cysts in humans. Finally, adverse events such as pain, pancreatitis, or infections remain a major concern in EUS-FNA procedures. Given the limitations of our animal model, further clinical trials are required to assess these adverse events.

On conclusion, the present study shows that the loop brush is apparently safe, capable of detaching cells from cavities in living organisms, and fully adaptable to the EUS-FNA procedure with a 22-gauge needle. In future work, we plan to assess the safety and efficiency of the loop brush with EUS-FNA for PCLs in a pilot clinical trial.

## Disclosure

The following author received research support for this study from The Swedish Cancer Society (19 0513 Fk 01 H): U. Arnelo. In addition, the following authors disclosed financial relationships: F. Marques, W. van der Wijngaart, N. Roxhed, F. Baldaque-Silva: Patent application for the loop brush; co-founders of Lucky Loop Medical AB. U. Arnelo: Patent application for the loop brush; co- founder of Lucky Loop Medical AB; research grant from Boston Scientific; consultant for Boston Scientific, Medtronic, and Ambu. All other authors disclosed no financial relationships. Research support for this study was also provided by Region Stockholm (HMT project no. 20180849) and MedTechLabs.

## Supplementary data

Appendix 3

Video 1 EUS-FNA procedure with a loop brush in an artificial cyst synchronized with the operator's perspective of the loop brush procedure. Videos accelerated 1.5 times.

Video 2 EUS-FNA with the loop brush in an artificial cyst including the following steps: cyst puncture, brush introduction into the cyst, brush rotation, brush removal from the cyst, aspiration of the liquid content of the cyst and needle removal from the cyst.
