# Supplementary material for: New through-the-needle brush for pancreatic cyst assessment: a randomized controlled trial
Source: IGIE. 2023 Aug 29;2(4):481–8. doi: 10.1016/j.igie.2023.08.006 (PMC12850763; doi:10.1016/j.igie.2023.08.006)
Supplement: Appendix 1 [file mmc1.pdf]

Appendix

Title:

New through-the-needle brush for pancreatic cysts assessment: a randomized control trial

Authors:
Filipe Marques,† Igor Schliemann,‡ Wouter van der Wijngaart,†
Urban Arnelo,¶,‖ Niclas Roxhed∗,†,⊥ and Francisco Baldaque-Silva∗,#,§,@

†KTH Royal Institute of Technology, Micro and Nanosystems, Malvinas v ̈ag 10, 100 44 Stockholm, Sweden
‡Pathology and Cytology Department, Karolinska University Hospital Stockholm, Sweden
¶Department of Surgical and Perioperative Sciences/Surgery, Umeå University, Umeå, Sweden
‖Division of Surgery, CLINTEC, Karolinska Institute, Stockholm, Sweden
⊥MedTechLabs, Bioclinicum, Karolinska University Hospital, Solna, Sweden
@Advanced Endoscopy Center Carlos Moreira da Silva, Gastroenterology Department, Pedro Hispano Hospital, Matosinhos, Portugal
#Department of Medicine, Karolinska Institute, Stockholm, Sweden
§Center for Upper Gastrointestinal Diseases, Karolinska University Hospital, Stockholm, Sweden

Correspondence to:
Niclas Roxhed (roxhed@kth.se), Francisco Baldaque-Silva (fbaldaquesilva@gmail.com)

Table of contents

1. EUS-FNA and artificial cyst preparation
2. Cytological and histological analysis
3. Hemoglobin and cell counting analysis
4. Safety tests
5. Cytological images
6. Diagnostic assessment, hemoglobin analysis and cell counting results

1. EUS-FNA and artificial cyst preparation

Pigs were pre-medicated with sedatives (intramuscular injection of tiletamine 2.5 $mg \cdot kg^{-1}$, zolazepam 2.5 $mg \cdot kg^{-1}$, medetomidine 0.1 $mg \cdot kg^{-1}$), taken to a fully equipped operating room, intubated and mechanically ventilated while receiving standard surgical anesthetic care, including the administration of heparin. Anesthesia was induced with i.v. propofol (20 mg) or i.v. sodium pentobarbital (120–180 mg) and maintained with sodium pentobarbital infusion (15–20 $mg \cdot kg^{-1} \cdot h^{-1}$). Analgesia was provided using i.v. fentanyl (50 $\mu g \cdot h^{-1}$). The pigs were monitored throughout the intervention using ECG, invasive blood pressure measurement, oxygen saturation, temperature, and urine production. EUS-FNA was surgically prepared in the following manner; Surgery began by performing an upper midline incision. The stomach was exposed and and following placement of hold sutures an about 10 cm long gastrotomy was made on the lower anterior side of the stomach. The stomach was emptied by manually removing large sustenance remnants, followed by irrigation/aspiration cycles with lukewarm water until the fluid was visibly clear. The stomach was closed and visually inspected using a gastroscope (GIF XP260, Olympus, Japan) and the Guardus® gastric overtube (Steris™, OH, USA) was positioned with the distal end reaching the lower part of the esophagus.

Artificial cysts were surgically prepared using small intestine sections, originating from the duodenojejunal flexure (localized via ligament of Treitz) to the jejunum onward. Each section was isolated using vessel loops distanced apart 20 cm, with two incisions at the proximal and distal endings.

Cleaning was then performed by repeatedly flushing lukewarm water through the proximal to the distal end of the section until the fluid was visibly clear. Excess fluid present inside the section was manually pushed out by massaging the tissue. Then, incisions were performed every 2 cm of the mesentery, allowing blood supply to a cyst through at least one jejunal artery and vein. Cable ties (36-7893, Clas Ohlson, Sweden) were inserted

through the incisions, enclosing the artificial cysts. The cysts were filled with approximately 5 mL of lukewarm water, using a 27G needle and were then anchored to the lower backside of the stomach by sutures. Cysts were cut open if any signs of strangulation were observed. The abdominal cavity was temporally closed during EUS-FNA. If needed, the procedure was repeated to produce all the cysts planned for that pig. In pig 1, the last two cysts (one control and one brushing) were collected for histology.

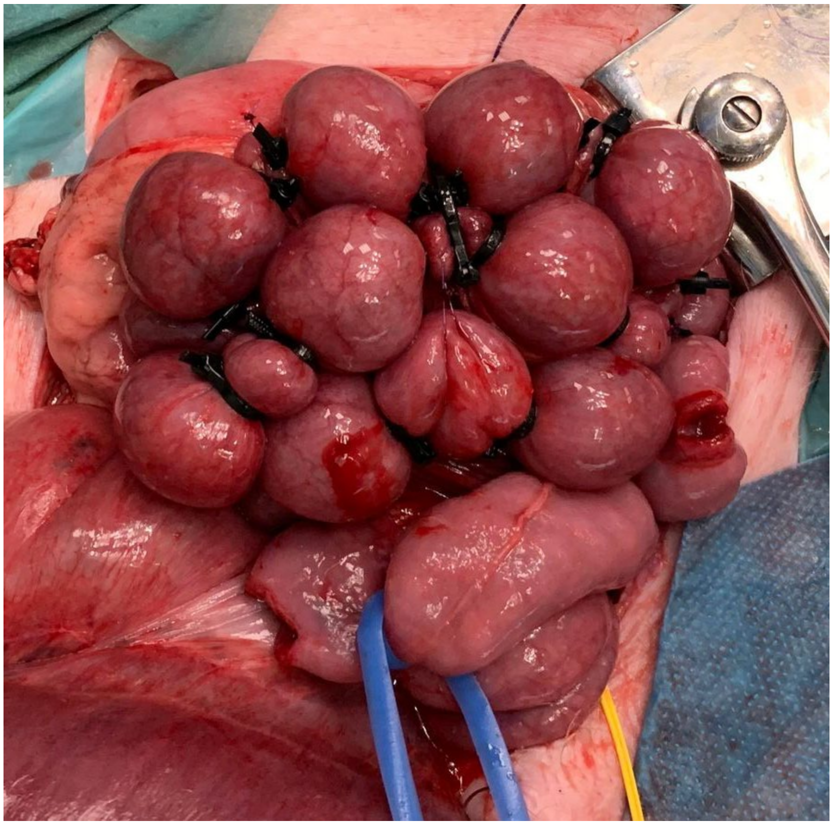

Figure 1: Picture of man-made cysts produced from the small intestine sutured to the antrum and body of a pig stomach. Two cysts were undone due to strangulation during cyst preparation.

2. Cytological and histological analysis

Following each procedure, a few droplets of sample were immediately smeared and stained using standard ROSE technique. Samples from pig 6 provided high yields of sustenance remnants; hence, could not be used for cell counting. The last two cysts from pig 1 were fixated in 4% formalin, placed in parafin blocks and sliced at 4 µm. The tissue was stained with H&E following common procedure and visually inspected using 4× and 10× objectives. Images were acquired with a E31SPM06300KPB (Sony, Japan) camera and analyzed with ImageView software (version 3.7.13777.20190114).

3. Hemoglobin and cell counting analysis

The remaining samples were placed in 15 mL centrifuge tubes (21008-105, VWR, Sweden) with 1 ml of PreservCyt (Hologic, MA, US) for transport. During sample preparation, samples were gently homogenized in 2 s aspiration/dispensing cycles with a pipette for 1 min. Hemoglobin analysis was performed using 100 μL of sample with a HemoCue® Hb 201+ System (HemoCue AB, Sweden). For cell counting, samples were again homogenized, and a 1 mL alliquote was placed in an Eppendorf tube and mixed with Hoechst 33342 (H3570, Thermo Fisher Scientific, Sweden) at a concentration of 0.05% v/v. After 20 min, 10 μL of sample was placed in a Countess™ cell counting chamber slide (C10228, Thermo Fisher Scientific, Sweden). Cell counting was achieved with a Countess II FL automated cell counter (Thermo Fisher Scientific, Sweden), coupled with an EVOS™ light cube, DAPI (AMEP4650, Thermo Fisher Scientific, Sweden). Cell counting settings were the following: size between 4-14 μm, brightness between 100-255 a.u., circularity of 0.67, and using autofocus. The initial volume of each sample was measured and both hemoglobin and cell count results are shown considering PreservCyt dilution.

4. Safety tests

The loop brush procedural safety was further tested in two more pigs with a total of 20 cysts. Artificial cysts and EUS-FNA with the loop brush using polyimide tubing were replicated as in the RCT, without controls and ROSE analysis. Loop brushes were again visually inspected and scrutinized for damage after each procedure and deemed successful following the same criteria described in the RCT. Hemoglobin, a surrogate marker for bleeding, in the cystic fluid was also analyzed. Adverse events were considered as described in the RCT. Safety tests data was compiled with RCT data. Statistical analysis followed the same methodology as in the RCT (see Annex 6).

There were no adverse events. Hemoglobin results showed that 75% (15/20) of the samples had non-detectable hemoglobin and the highest concentration was 0.7 g/dL. Cell concentrations of samples were in the same order of magnitude as samples in the RCT.

5. Cytological images

Cytology of loop-brush samples indicated the presence of cells, cell clusters (Figure 2a-b), mucus (Figure 2c) and sustenance remnants (Figure 2d). Cells remained intact and could be used to provide a diagnosis. However, in cases where large quantities of sustenance were present in samples, there were difficulties in providing a diagnosis. In these cases, cells would be enveloped in sustenance and hinder analysis. This shows an obvious weakness of our model since sustenance is not expected in PCLs.

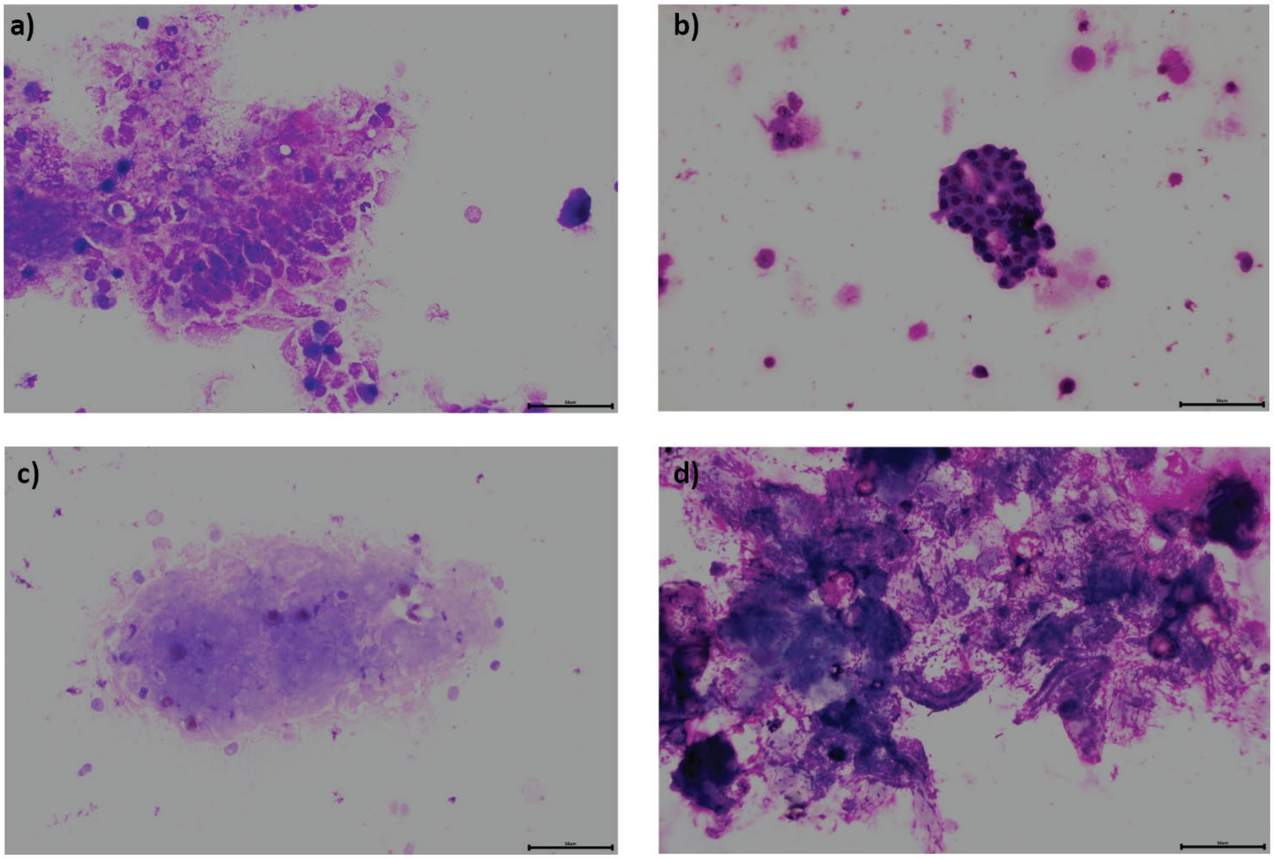

Figure 2 Cytological pictures of loop-brush samples from man-made cysts. a) Enterocytes. b) Enterocyte cluster and individual enterocytes. c) Mucus. d) Sustenance remnants.

6. Diagnostic assessment, hemoglobin analysis and cell counting results

Table 1: Results overview of controls and loop-brushes during a pre-clinical assessment in man-made cysts.

Pig #	Control					Loop brush					
	n (cyst)	Possible to diagnose	Diagnostic Percentage (%)	Average cell counting (cells/ mL)	Average hemoglobin (g/dL)	*n*	Possible to diagnose	Diagnostic Percentage (%)	Average cell counting (cells/ mL)	Average hemoglobin (g/dL)	Comments
1	8	7	88	430 000	0	8	8	100	1 920 000	0	No adverse events
2	9	4	44	74000 (n=7)	0.1	9	5	55	1 540 000	0	1 Teflon tube broke during removal from the cyst. No adverse events
3	10	8	80	460 000	0	10	10	100	1 630 000	0	No adverse events
4	9	3	33	112 000	0	9	7	78	1 710 000	0	1 Device failed to be pushed into a cyst. No adverse events
5	9	4	44	1 350 000	0	9	6	66	4 810 000	0	No adverse events
6	12	4	33	N/A (Excluded due to sustenance)	0.25	12	8	83	N/A (Excluded due to sustenance)	0.2	1 Teflon tube broke during removal from the cyst. No adverse events
7	N/A	N/A	N/A	N/A	N/A	10	N/A	N/A	11 800 000	0.1	No adverse events
8	N/A	N/A	N/A	N/A	N/A	10	N/A	N/A	5 040 000	0.1	No adverse events

An overview of all analysis performed during the RCT (pigs 1 to 6) and safety tests (pigs 7 and 8) with respect to controls and loop brushes can be seen in Table 1. It details the number of cysts used on each group, the diagnostic assessment and percentage, average cell counting concentration and average hemoglobin concentration per animal.

Hemoglobin analysis of all samples in the RCT and safety tests show a non-significant difference (p=0.32) between no brushing and brushing (Figure 3a). Cell count results show a significant difference between no brushing and brushing, in all pigs (including pigs 7 and 8) and for pigs 1 to 5 with p-values of <.0001, .0005, <.0001, .0004, .0094, and .0039, respectively. Cell concentrations of samples in pigs 7 and 8 were in the same order of magnitude as samples in the RCT (Figure 3b).

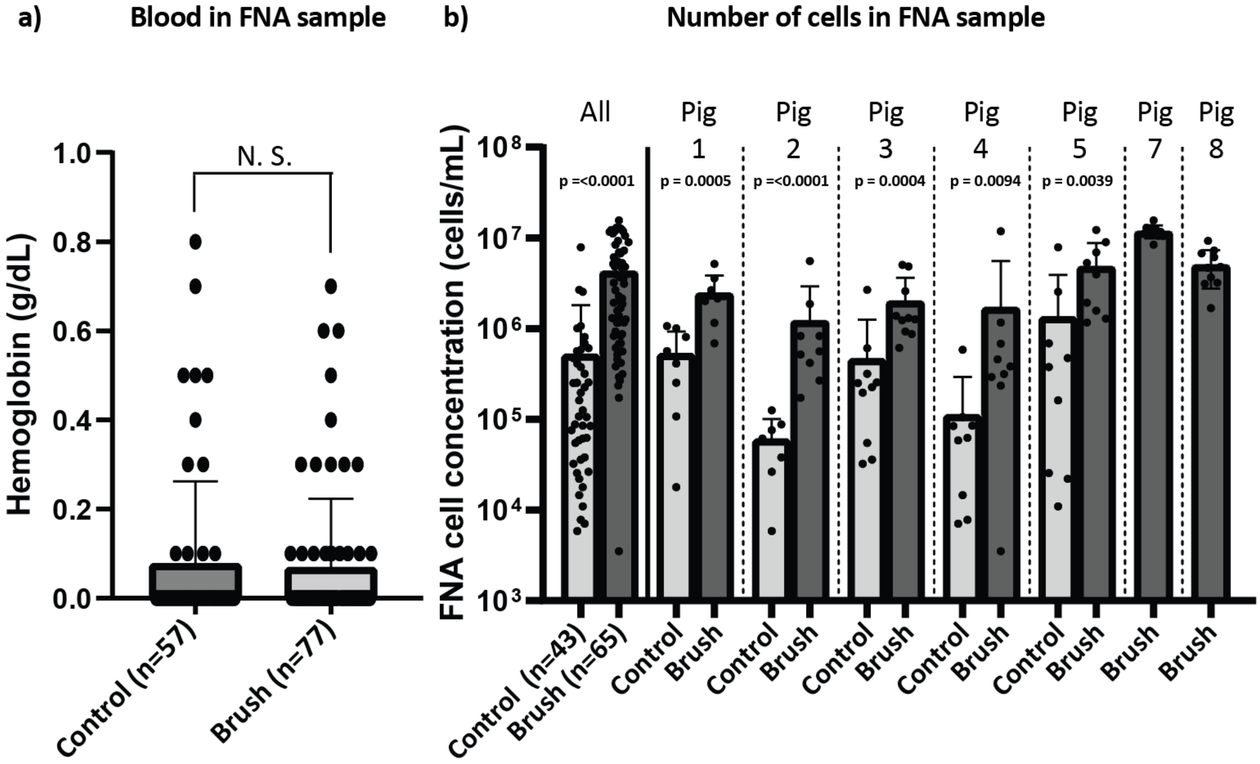

Figure 3 Pre-clinical assessment of the loop brush in artificial cysts including RCT and safety tests data. **a)** Hemoglobin analysis of 57 controls and 77 loop brush fine needle aspiration samples showing no significant difference between controls and brushings (p=0.32). **b)** Cell count results of controls and loop brushings from all pigs (including pigs 7 and 8) and pigs 1 to 5 with p-values of <.0001, .0005, <.0001, .0004, .0094, and .0039, respectively, indicating a significant difference between brushing and no brushing. Pig 6 and two samples in pig 2 were excluded due to excess sustenance in samples. No controls were performed in pigs 7 and 8
