# Supplementary material for: New through-the-needle brush for pancreatic cyst assessment: a randomized controlled trial
Source: IGIE. 2023 Aug 29;2(4):481–8. doi: 10.1016/j.igie.2023.08.006 (PMC12850763; doi:10.1016/j.igie.2023.08.006)
Supplement: Appendix 3 [file mmc3.pdf]

GIE IRB Checklist

Note that GIE follows the International Committee of Medical Journal Editors (ICMJE)'s Uniform Requirement for Manuscripts Submitted to Biomedical Journals. All clinical trials submitted to GIE should have been registered BEFORE the trial begins through one of the registries approved by the ICMJE, and proof of that registration, including the date registered and the registration number, must be submitted to GIE along with the article. IRBH approval information must be included in the manuscript text, including the date of registration. All clinical trials as defined by the ICMJE must also have been registered before the trial began (not just randomized clinical trials).

Yes	Have you included IRB information in your article? If not, please explain why. Do not merely put NA or your article will be returned for a fuller explanation.<br>Comments:
15/01/2021	Please list here the DATE of IRB approval for your study.
Animal trial<br>NA	Proof of registration for randomized clinical trials, including registration number and dates of when patients were enrolled, when trial was registered, and when the trial was started, is required before subject enrollment; have you included this information?
